# Volumetric Thermal Characterisation of a Controlled Bioprinting Chamber Using Multi-Point Temperature Sensing

**DOI:** 10.3390/biomimetics11080552

**Published:** 2026-08-04

**Authors:** Alfonso C. Marcos-Romero, Manuel Matamoros-Pacheco, Laura Mendoza-Cerezo, Silvia M. Díaz-Prado, Jesús M. Rodríguez-Rego

**Affiliations:** 1 Departamento de Expresión Gráfica, Escuela de Ingenieros Industriales, Universidad de Extremadura, Avenida de Elvas, s/n., 06006 Badajoz, Spainlmencer@unex.es (L.M.-C.); 2 Departamento de Bioquímica, Biología Molecular y Genética, Facultad de Ciencias, Universidad de Extremadura, Avenida de Elvas, s/n., 06006 Badajoz, Spain; 3 Grupo de Investigación en Terapia Celular y Medicina Regenerativa, Instituto de Investigación Biomédica de A Coruña (INIBIC), Universidade de A Coruña, Complexo Hospitalario Universitario de A Coruña (CHUAC), Servizo Galego de Saúde (SERGAS), 15006 A Coruña, Spain; 4 Centro de Investigacións Científicas Avanzadas (CICA), Universidade da Coruña (UDC), 15008 A Coruña, Spain; 5 Departamento de Medicina, Facultad de Ciencias de la Salud, Universidade da Coruña (UDC), 15006 A Coruña, Spain; 6 Centro de Investigación Biomédica en Red de Bioingeniería, Biomateriales y Nanomedicina (CIBER-BBN), 28029 Madrid, Spain

**Keywords:** 3D bioprinting, thermal distribution, atmospheric chamber, experimental characterisation, computational fluid dynamics

## Abstract

3D bioprinting requires control of environmental conditions within the printing chamber, as temperature affects bioink rheology, printability and cell viability. However, the spatial temperature distribution inside bioprinting enclosures remains poorly characterised, limiting the understanding of thermal gradients that may affect process stability. In this work, the spatial thermal behaviour of a previously developed controlled chamber was evaluated using a multi-point temperature acquisition system. Temperature was monitored at 45 locations distributed throughout the chamber volume under controlled conditions at 37 °C after thermal stabilisation. The results revealed vertical and lateral thermal gradients associated with natural convection and forced air recirculation, together with local non-uniformities influenced by fan operation. Nevertheless, comparatively homogeneous temperature regions were identified within the printing zone, indicating suitable areas for more stable and reproducible biofabrication processes. Additionally, a three-dimensional CFD model incorporating the internal air volume, two 200 W electrical heaters, two axial recirculation fans, and simplified representations of the printhead and build platform was developed to represent an operational chamber configuration. The model was used to visualise the spatial temperature distribution within the enclosure, including the thermal field around the internal printer components. The proposed approach provides a practical experimental framework for the volumetric characterisation of thermal conditions in bioprinting environments, contributing to the design and optimisation of controlled chambers and improving the reliability of biofabrication processes.

## 1. Introduction

3D bioprinting has become one of the most promising technologies in the field of biomedical engineering, enabling the fabrication of complex and biomimetic tissue constructs through the controlled deposition of cell-laden materials [1,2]. Its applications range from the development of models for drug testing to tissue engineering for regenerative medicine [3,4]. In these applications, the objective is not limited to reproducing the external geometry of a tissue, but also to generating constructs that recreate relevant aspects of the native cellular microenvironment, including hydration, mechanical response, matrix-like architecture and biological functionality. This biomimetic perspective is one of the main reasons why hydrogel-based bioinks have become central to the development of advanced biofabrication strategies.

However, obtaining functional and reproducible biomimetic structures remains a challenge, due to the interaction of multiple physical, chemical and biological factors that must be carefully controlled. The fabrication of these constructs does not depend exclusively on the formulation of the bioink or on the nominal printing parameters. The environmental conditions inside the printing chamber can also influence the behaviour of cell-laden materials during deposition and early stabilization. Temperature is especially relevant in this regard, since many hydrogel-based bioinks present temperature-dependent rheological or gelation behaviour. Spatial thermal variations within the chamber may therefore affect viscosity, filament formation, construct stability and cell response, ultimately influencing the reproducibility of biomimetic tissue models and engineered constructs.

Hydrogels are the most widely used materials in bioprinting due to their high-water content and their similarity to the extracellular matrix [5]. However, their physicochemical and rheological properties are highly sensitive to environmental conditions. In particular, temperature plays a critical role in bioink viscosity, gelation processes and cell viability during and after the printing process. Even moderate thermal variations may affect printing fidelity, the structural stability of the construct and its biological behaviour [5,6,7].

Current commercial bioprinters usually incorporate thermal control systems located in the extrusion head or on the printing platform, but they do not guarantee a homogeneous temperature distribution throughout the chamber volume. This limitation is particularly relevant during the post-printing stage, where uncontrolled thermal gradients may induce dehydration, material contraction or heterogeneous cellular behaviour [8,9,10]. Although some studies have addressed the integration of environmental control systems in bioprinting, these approaches mainly focus on modelling and controlling local temperatures, such as those of the nozzle, platform or microchannels, without analysing in detail the thermal distribution within the chamber volume [11,12,13].

The design and optimisation of controlled atmospheric enclosures for bioprinting remains an insufficiently addressed problem. In related fields, the importance of thermal uniformity and airflow patterns has been demonstrated by combining experimental measurements and computational fluid dynamics (CFD) simulations to analyse heat transfer phenomena [14,15,16]. However, these approaches have not been directly translated to the field of bioprinting, where the operating conditions, system geometry and material sensitivity present specific characteristics.

In this context, the present work combines the experimental characterisation of the thermal behaviour of a previously developed atmospheric enclosure for 3D bioprinting with a three-dimensional CFD analysis. A data acquisition system capable of monitoring multiple points inside the chamber was implemented, allowing the spatial temperature distribution to be analysed under controlled conditions. The CFD model included the main chamber geometry, thermal-conditioning components and simplified stationary adiabatic representations of the printhead and build platform. The numerical analysis was used to visualise the spatial temperature field within an operational chamber geometry accounting for the geometric presence of these internal printer components. The aim was to provide an experimental and numerical basis for analysing thermal behaviour in bioprinting environments and to contribute to the optimisation of this type of system.

Although thermal gradients in heated enclosures are expected from basic heat-transfer mechanisms, their spatial distribution within the working volume of bioprinting chambers is rarely characterised experimentally. In most bioprinting systems, thermal performance is commonly assessed through nominal chamber setpoints, local control points or numerical estimations, which do not necessarily describe the conditions experienced across the complete deposition region. In this work, a multi-point experimental approach is used to map the temperature distribution within the working volume of a controlled bioprinting chamber, allowing spatial gradients, lateral asymmetries and comparatively stable regions to be identified under defined operating conditions. The numerical analysis additionally visualised the temperature field after incorporating the printhead and build platform into the computational domain as stationary adiabatic surfaces.

## 2. Materials and Methods

### 2.1. Atmospheric Chamber

The atmospheric enclosure used in this study was previously developed and validated for temperature and humidity control in 3D bioprinting applications [10]. While the previous work focused on the general temperature and humidity control capability of the enclosure, the present study addresses the spatial distribution of temperature within the chamber working volume. The present multi-point volumetric assessment characterises internal thermal gradients, lateral asymmetries and comparatively stable regions relevant to biofabrication conditions. The chamber features directly influencing its thermal behaviour are described below.

The system consists of a closed chamber in which temperature is generated by electrical heating elements located in a dedicated sub-chamber. Airflow is driven by axial fans that continuously recirculate air through the system, promoting forced convection. This configuration establishes a coupled heat transfer mechanism governed by both forced airflow and natural convection effects, which directly determine the spatial temperature distribution.

The chamber incorporates a secondary compartment where air is conditioned before being reintroduced into the main volume, forming a continuous recirculation loop. The interaction between airflow patterns and heat sources leads to the development of thermal gradients within the enclosure.

**Figure 1:**
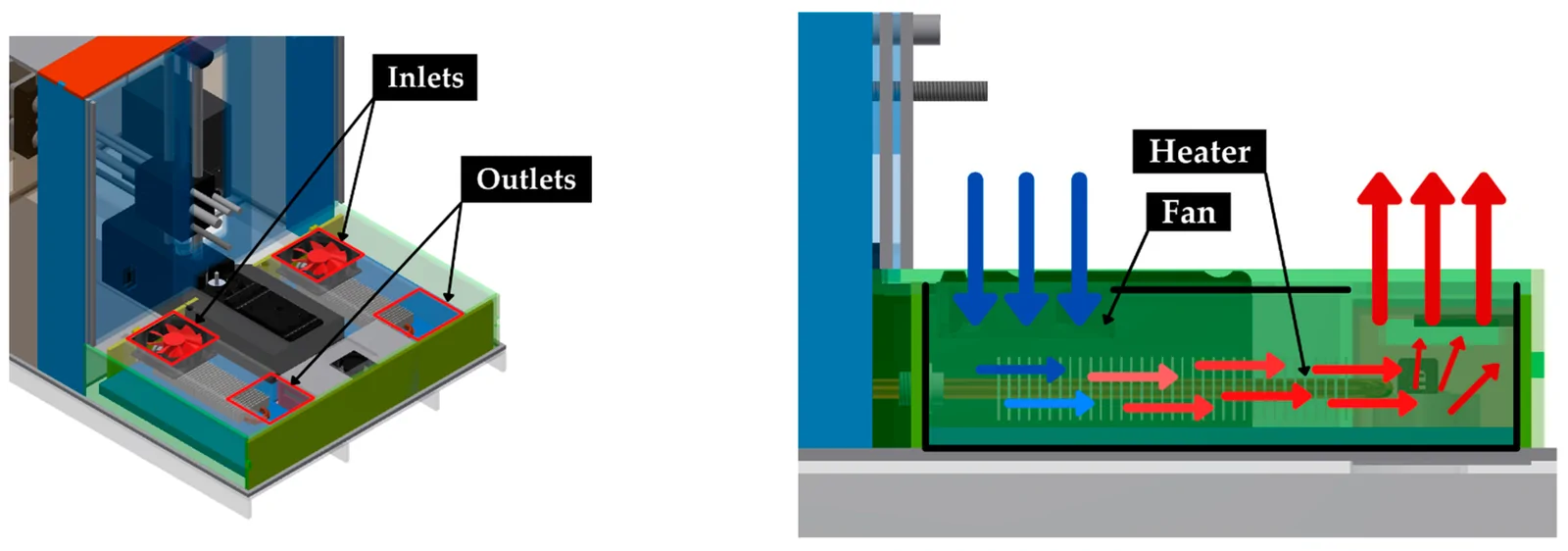
Schematic representation of the airflow configuration inside the atmospheric chamber, showing air inlets, outlets, heating elements and forced convection patterns.

The airflow configuration, including the location of inlets, outlets, heating elements and fans, is schematically shown in Figure 1. This arrangement defines the internal circulation patterns that govern temperature distribution throughout the chamber.

The chamber geometry and the relative position of heating and airflow components were maintained as defined in the original system design [10], ensuring consistent operating conditions during all experiments. These parameters are critical for the analysis of temperature gradients and thermal homogeneity within the printing environment.

The positioning system was based on an open-source Proton3D printer. Owing to its mechanical architecture, only the printhead and build platform are located inside the thermally controlled enclosure, whereas the remaining positioning and structural components remain outside the chamber. In the CFD model, the printhead and build platform were represented as stationary adiabatic surfaces reproducing their geometric presence within the chamber. Their inclusion therefore accounted for their influence on the available airflow domain and local air-recirculation patterns.

### 2.2. Temperature Acquisition System

A multi-point temperature acquisition system was developed to enable the spatial characterisation of thermal conditions within the atmospheric chamber. The system is based on a modular sensor frame designed to measure temperature at multiple locations within a defined cross-sectional area of the chamber.

**Figure 2:**
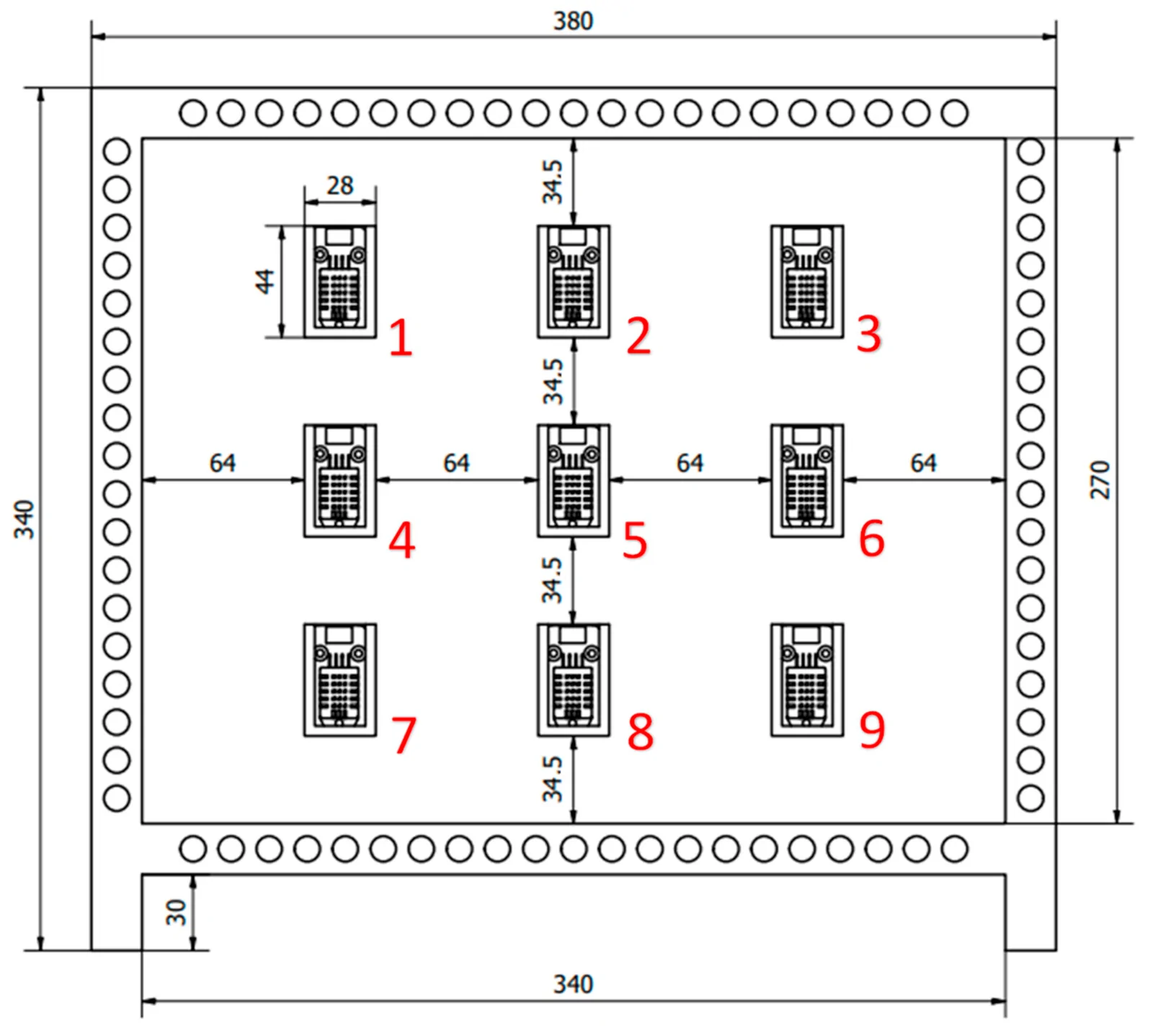
Temperature sensor frame measurements.

The acquisition system consists of an array of 9 digital temperature sensors (DHT22, Cetronic S.L., located in A Coruña, Spain) mounted on a rigid frame matching the dimensions of the chamber cross-section. The sensors were arranged in a regular 3 × 3 grid configuration, ensuring uniform spatial coverage of each measurement plane. The geometry and dimensions of the sensor frame are shown in Figure 2.

The DHT22 sensors provide temperature measurements within a range of −40 °C to 80 °C, with an accuracy of ±0.5 °C. Temperature data were recorded at approximately 2.5 s intervals using a centralized data acquisition system. This sampling interval was maintained during the acquisition period performed after thermal stabilization.

The DHT22 sensors, with a nominal accuracy of ±0.5 °C, enabled the identification of the main spatial thermal trends within the chamber. Measurement uncertainty was considered during data processing and interpretation, as described in Section 2.4 and Section 3.1.

To achieve volumetric characterisation of the chamber, the sensor frame was used to perform measurements at multiple locations by sequentially positioning it along different sections of the enclosure. This approach allows reconstructing the three-dimensional temperature distribution using repeated measurements across the chamber volume.

A calibration procedure was performed prior to experimentation to ensure consistency among sensors. All sensors were placed in a controlled environment at constant temperature and positioned at the same height to avoid stratification effects. Deviations between sensors were quantified and corrected by applying individual offsets, improving the accuracy and comparability of the measurements.

**Figure 3:**
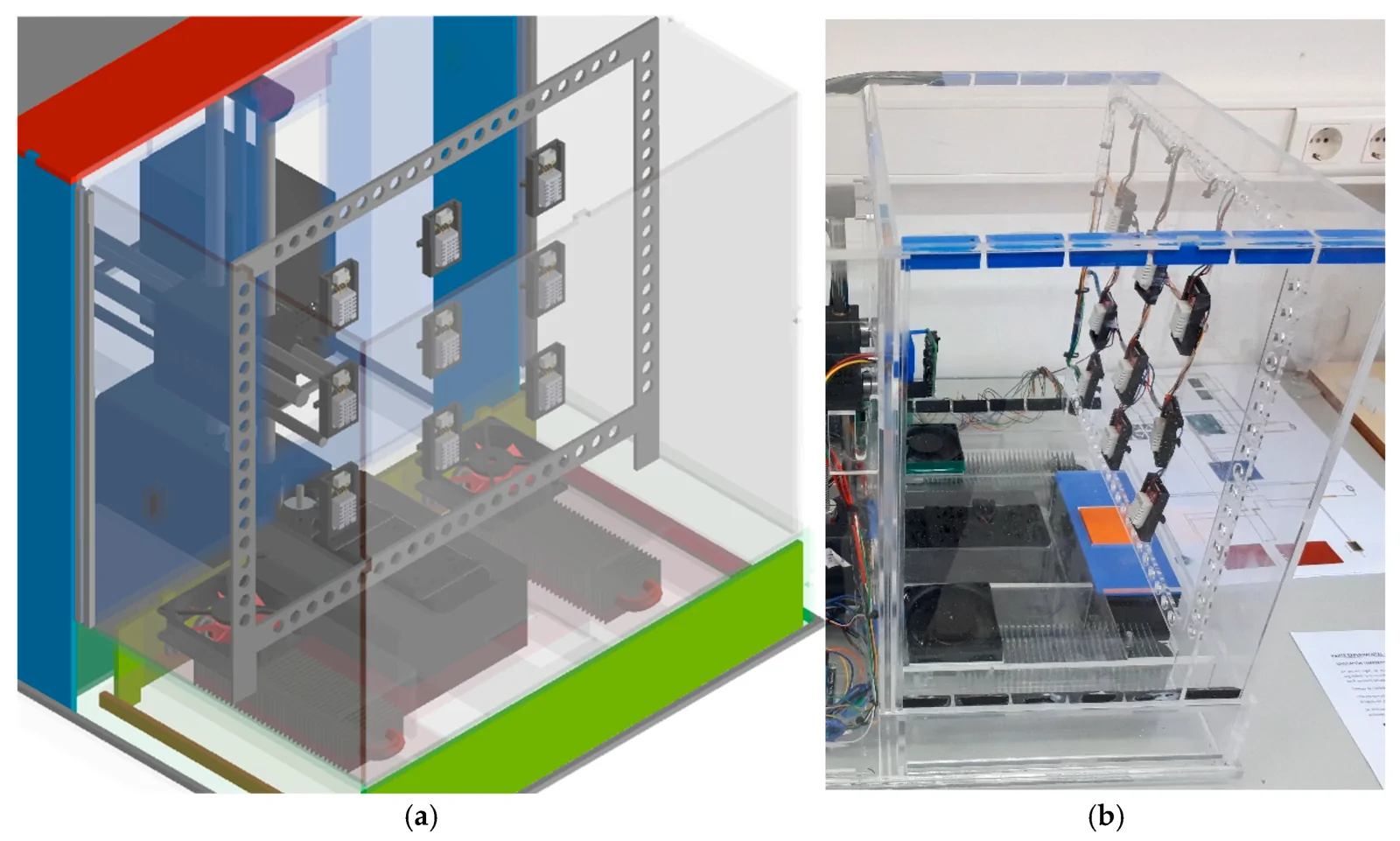
Rendering (**a**) and real image (**b**) of the sensor array inside the bioprinting sub-chamber.

A schematic representation of the sensor array and its integration within the chamber is shown in Figure 3, including both the CAD rendering and the real experimental setup. This configuration enables capturing temperature variations in regions influenced by airflow patterns and heating elements, supporting detailed analysis of thermal gradients and temperature homogeneity within the enclosure.

### 2.3. Experimental Procedure

Thermal characterisation experiments were conducted to evaluate the spatial temperature distribution within the bioprinting sub-chamber under controlled conditions. The internal temperature of the chamber was set to 37 °C, representative of standard bioprinting conditions, while the ambient temperature was maintained at approximately 25 °C.

**Figure 4:**
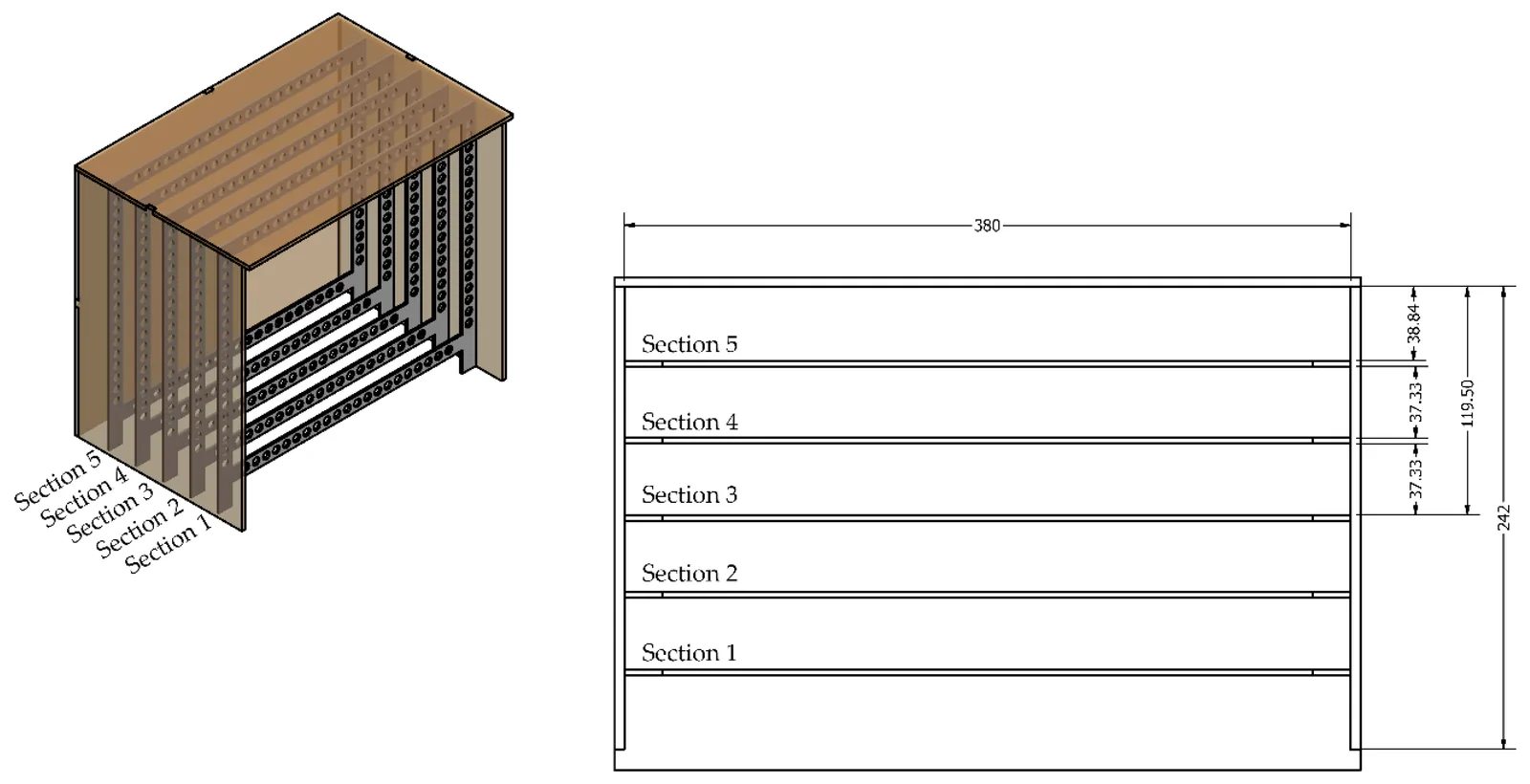
Placement of the sensor frame in bioprinting sub-chamber.

The temperature acquisition system described in Section 2.2 was used to perform measurements at different locations within the chamber. The sensor frame was sequentially positioned in five planes along the chamber depth, as illustrated in Figure 4, enabling volumetric characterisation of the temperature field.

For each section, the chamber was first allowed to reach steady-state conditions from ambient laboratory temperature. Temperature stabilization was considered achieved when the temporal variation in the measured values remained within ±0.5 °C over 10 min. Under the tested operating conditions, the chamber required approximately 45 min to reach stable operation before the 10 min acquisition period was started. Once stabilization was achieved, temperature data were recorded simultaneously at the 9 sensor positions during the acquisition period.

This acquisition period was used to compare the stabilised spatial temperature distribution among the five chamber sections.

This measurement procedure was repeated three times for each section to ensure reproducibility and reduce experimental variability. All experiments were conducted under identical operating conditions, maintaining the same heater-control strategy and airflow configuration throughout the tests.

Prior to the experimental campaign, a calibration process was carried out for both the chamber sensors and the sensors integrated in the acquisition frame. Calibration was performed in a controlled environment at constant temperature, with all sensors positioned at the same height to avoid stratification effects. Sensor deviations were quantified and corrected by applying individual offsets, improving the accuracy and consistency of the measurements.

The experimental reference configuration comprised the chamber, sensor frame and intrinsic mechanical and electronic components of the enclosure. This configuration was used to characterise the thermal field generated by the chamber geometry, heating system and air-recirculation arrangement. The numerical geometry subsequently incorporated simplified stationary adiabatic representations of the printhead and build platform to reproduce their geometric presence within the airflow domain.

### 2.4. Data Processing

The temperature data acquired during the experimental procedure were processed to evaluate spatial temperature distribution and thermal homogeneity within the chamber. For each section λ = 1, …, 5 and each sensor position *n* = 1, …, 9, the temperature values recorded during the acquisition period after thermal stabilization were first averaged within each experimental repetition. The final value assigned to each sensor position corresponded to the average of the three independent repetitions performed under identical operating conditions. The standard deviation between repetitions was used to describe inter-repetition variability and was represented as error bars in the corresponding graphs.

$${\bar{x}}_{\left({Section}_{\gamma },{Sensor}_{n}\right)}=\frac{1}{3}\;\sum_{i=1}^{3}\bar{x}\left(S_{n},T_{i}\right)=\frac{{\bar{x}}_{\left(S_{n},T_{1}\right)}+{\bar{x}}_{\left(S_{n},T_{2}\right)}+{\bar{x}}_{\left(S_{n},T_{3}\right)}}{3}$$

For each chamber section, the resulting mean temperature values were used to compare the spatial distribution across the nine sensor positions. These processed data enabled the identification of vertical gradients, lateral asymmetries and regions with comparatively more homogeneous thermal conditions. The same values were also used to generate the spatial temperature maps for each measurement section, allowing comparison between the different planes of the chamber.

Descriptive and statistical analyses were performed using Microsoft Excel. Spatial differences among the nine sensor positions were assessed using the Friedman test. Kendall’s coefficient of concordance (W) was also calculated to evaluate the consistency of the spatial temperature pattern. Statistical significance was set at *p* < 0.05.

The selected spatial resolution provided a discrete volumetric characterisation of the main thermal trends within the chamber. The 3 × 3 sensor array allowed comparison between the upper, central and lower regions of each measurement plane, while its sequential positioning in five sections enabled these patterns to be assessed along the chamber depth.

The nominal accuracy of the DHT22 sensors was considered when interpreting the measured temperature differences. The analysis focused on repeated spatial patterns, section-level temperature ranges and trends consistent across experimental repetitions, with the nominal sensor accuracy of ±0.5 °C incorporated into the interpretation of local differences.

### 2.5. Rheological Assessment of a Temperature-Sensitive Hydrogel

To complement the thermal characterisation of the chamber and assess the potential relevance of the measured temperature gradients for temperature-sensitive hydrogel systems, rheological measurements were performed using a gelatin/DMEM hydrogel as a model thermoresponsive formulation. Gelatin was selected because its rheological behaviour is strongly temperature-dependent and because gelatin-containing formulations are commonly used as thermoresponsive model bioinks in extrusion-based bioprinting.

The hydrogel was prepared by dissolving gelatin at 10% (*w*/*v*) in PBS at 50 °C under continuous stirring until complete dissolution. The resulting gelatin hydrogel was subsequently mixed with complete DMEM at a 3:1 volume ratio, reproducing the dilution step that occurs when a hydrogel precursor is combined with a cell suspension prior to bioprinting.

Rheological measurements were performed using a rotational rheometer (Kinexus Pro+, Netzsch, Germany) equipped with a 40 mm parallel-plate geometry and a gap of 0.4 mm. Before each measurement, the sample was allowed to equilibrate for 15 min under the corresponding test conditions.

An oscillatory temperature sweep was conducted from 26 to 38 °C at a heating rate of 0.5 °C·min^−1^, using a constant strain of 0.5% and a frequency of 1 Hz. The storage modulus (G′) and loss modulus (G″) were recorded as functions of temperature during heating.

Apparent viscosity measurements as a function of shear rate were also performed at 31, 35 and 39 °C. At each temperature, the sample was allowed to equilibrate for 15 min before testing. Apparent viscosity was recorded during an ascending shear-rate sweep from 0.1 to 1000 s^−1^. A fresh sample was used for each temperature condition. All rheological measurements were performed in triplicate, and the results were expressed as mean ± standard deviation.

### 2.6. CFD Model

#### 2.6.1. Geometry and Numerical Configurations

A three-dimensional computational fluid dynamics model of the controlled bioprinting chamber was developed using ANSYS Fluent R16.2 (ANSYS Inc., Canonsburg, PA, USA). The numerical geometry was generated from the experimental enclosure and reproduced the internal air volume, the thermal-conditioning and recirculation regions, two electrical heaters, two axial fans, the air inlet and outlet regions, and the methacrylate chamber walls. Non-essential geometrical details were simplified to reduce the computational cost while preserving the main elements governing airflow and heat transfer.

**Figure 5:**
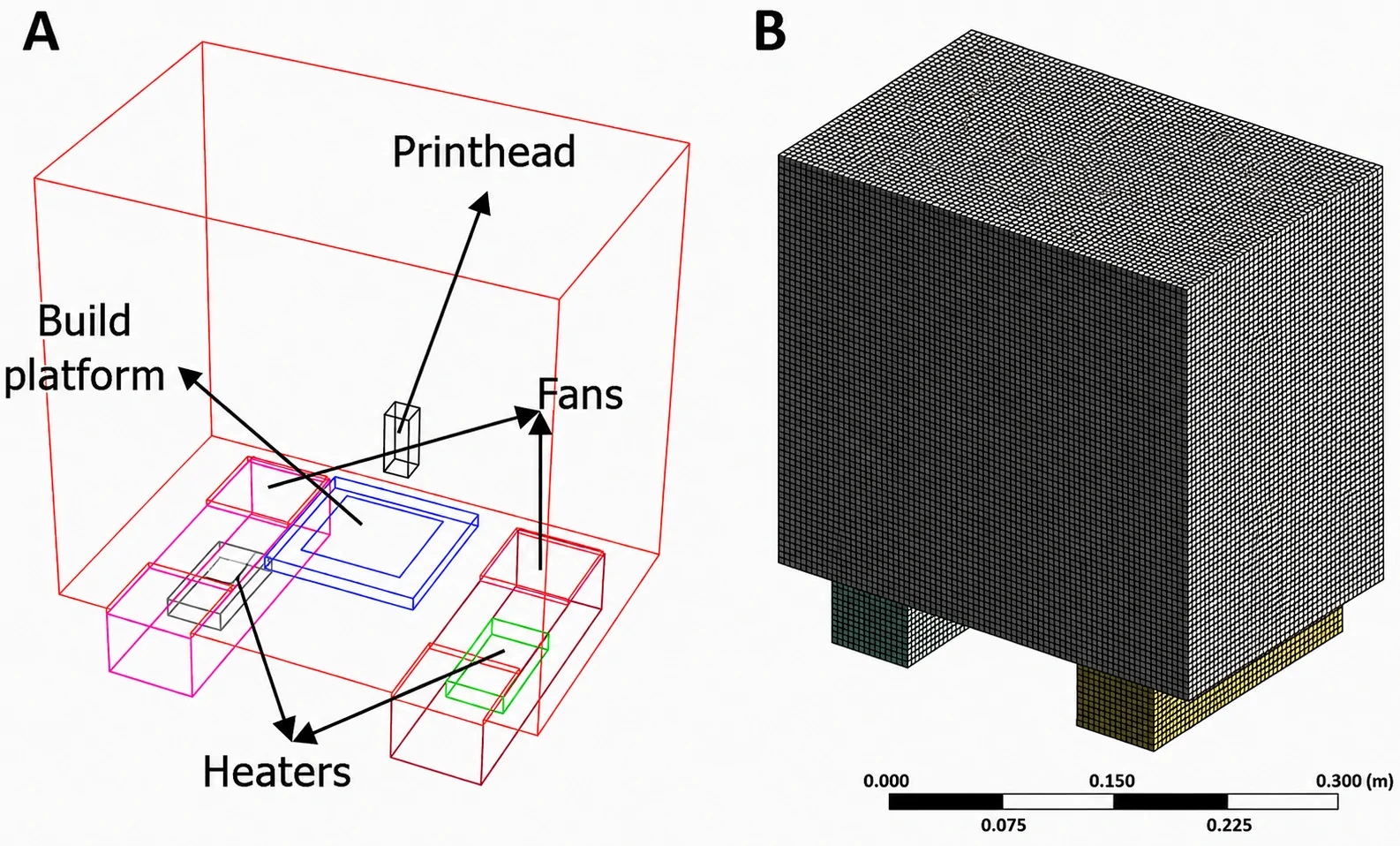
CFD geometry and computational mesh of the controlled bioprinting chamber. (**A**) Three-dimensional geometry including the chamber volume, thermal-conditioning regions, two electrical heaters, two axial fans, and simplified representations of the printhead and build platform. (**B**) Final unstructured computational mesh, including local refinement around the thermal-conditioning components and printing region.

Two numerical configurations were considered. The reference configuration reproduced the chamber arrangement used during the experimental temperature measurements. The operational configuration additionally incorporated simplified stationary adiabatic representations of the printhead and build platform at a representative printing position, as shown in Figure 5.

The printhead and build platform were represented by stationary adiabatic wall surfaces defining their external geometry within the computational domain. The printhead was represented with dimensions of 20 × 20 × 50 mm, whereas the build platform was represented with dimensions of 120 × 120 × 10 mm.

The printhead was located at the centre of the printing area. The upper surface of the build platform was positioned 20 mm above the chamber base, with a nozzle-to-platform distance of 30 mm. This arrangement represented the intermediate printing position analysed in the operational CFD configuration.

The chamber was integrated with an open-source Proton3D positioning system. Owing to the architecture of this printer, only the printhead and build platform were located inside the thermally controlled enclosure, whereas the remaining mechanical and structural components remained outside the chamber. The printhead and build platform therefore constituted the principal printer-related geometric obstacles represented within the airflow domain.

#### 2.6.2. Computational Mesh

The computational domain was discretised using an unstructured mesh composed predominantly of tetrahedral and hexahedral elements, with local refinement around the electrical heaters, axial fans, printhead, build platform and printing region. The final mesh contained 284,154 elements. The final computational mesh is shown in Figure 5B.

#### 2.6.3. Physical Model and Boundary Conditions

The conservation equations for mass, momentum and energy were solved throughout the computational domain. Air was defined as the working fluid using the thermophysical properties available in the ANSYS Fluent material database, and its density was calculated using the ideal-gas relationship:

$$\rho =\;\frac{p}{RT}$$

where *ρ* is the air density, *p* is the absolute pressure, *R* is the specific gas constant for air and *T* is the absolute temperature.

Gravity was applied in the vertical direction to represent buoyancy-driven flow together with the forced circulation generated by the fans.

The thermal model included forced convection, buoyancy-driven natural convection and heat conduction through the chamber walls. Thermal coupling was defined at the fluid–solid interfaces between the air domain and the chamber walls. The printhead and build platform were represented as stationary adiabatic wall boundaries, with zero heat flux imposed through their surfaces.

The enclosure walls were modelled as 5 mm thick polymethyl methacrylate solid domains. The material was assigned a density of 1180 kg·m^−3^, a specific heat capacity of 1460 J·kg^−1^·K^−1^ and a thermal conductivity of 5.3 W·m^−1^·K^−1^. Heat exchange between the external wall surfaces and the laboratory environment was represented by a convective boundary condition with an ambient temperature of 25 °C and a heat-transfer coefficient of 5.5 W·m^−2^·K^−1^.

The heating system consisted of two electrical heaters, each with a nominal thermal power of 200 W and a heat-exchange surface area of 0.01155 m^2^. The corresponding surface heat flux was calculated as:

$$q^{\prime \prime }=\frac{P}{A}$$

resulting in:

$$q^{\prime \prime }=\frac{200\;W}{0.0115\;m^{2}}=1.7316\times 10^{4}\;W/m^{2}.$$

The heaters were controlled using an on-off temperature-dependent condition based on the temperature at the control sensor location. The control temperature was evaluated at bioprinting zone, with a setpoint of 37 °C. The heaters were activated when the control temperature decreased below 37 °C and deactivated when it exceeded 37 °C.

The two axial fans were represented using the Fan Boundary Condition available in ANSYS Fluent. A pressure jump of 100 Pa was imposed across each fan in the direction indicated in Figure 1, reproducing the internal air-recirculation flow. The inlet and outlet regions of the recirculation compartment were defined according to the physical geometry of the chamber, and the internal interfaces were thermally and hydraulically coupled to the main air domain.

#### 2.6.4. Numerical Configuration and Temperature-Field Analysis

A transient pressure-based solver was used to calculate the airflow and temperature fields within the chamber. Turbulence was represented using the k-ω shear stress transport model. Pressure–velocity coupling was resolved using the SIMPLE algorithm, while the pressure, momentum, energy, turbulent kinetic energy and specific dissipation rate equations were discretised using Second Order schemes. Temporal discretisation was performed using a First Order Implicit formulation.

The air domain and the chamber-wall solid domains were initialised at 25 °C, with an initial air velocity of 0 m·s^−1^. The simulations were performed using a time step of 0.1 s, with a maximum of 20 iterations per time step and a total simulated time of 15 min. The time-step size was selected on the basis of numerical stability.

Convergence within each time step was established when the scaled residuals decreased below 0.001 for continuity, momentum, turbulent kinetic energy and specific dissipation rate, and below 1 × 10^−6^ for the energy equation. The volume-averaged temperature of the printing region and the mass-flow balance across the recirculation system were additionally monitored during the solution.

Thermal stabilisation was defined by a variation in the volume-averaged temperature of less than 1 °C over a period of 5 min. The reference and operational configurations were simulated using the same numerical settings and thermal-control conditions.

After thermal stabilisation, temperature contours were extracted from both numerical configurations. The reference configuration was used to visualise the temperature field generated by the chamber geometry, heating system and air-recirculation arrangement, whereas the operational configuration was used to visualise the temperature field with the stationary adiabatic representations of the printhead and build platform present within the airflow domain.

## 3. Results and Discussion

### 3.1. Experimental Results

**Figure 6:**
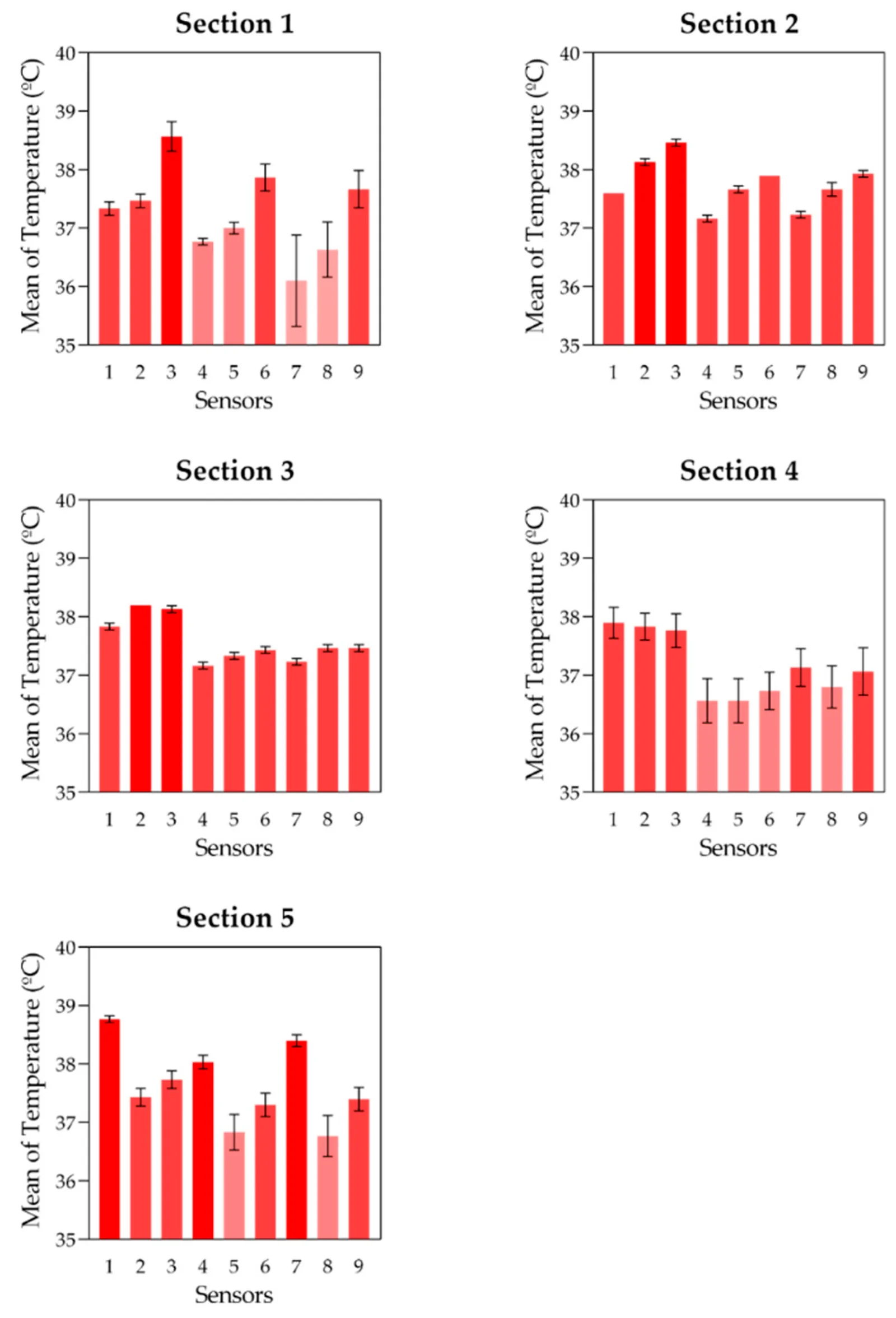
Mean temperature values recorded by each sensor in the five measurement sections of the bioprinting chamber after thermal stabilization. Error bars represent the variability between experimental repetitions.

The mean temperature values obtained for each sensor in the five sections of the chamber are presented in Figure 6. These results provide an initial comparison of the spatial temperature distribution within each measurement plane after thermal stabilization.

A consistent thermal pattern was observed across all sections, characterised by a vertical temperature gradient. Sensors located in the upper region of the chamber (positions 1, 2 and 3) systematically recorded higher temperatures than those located in the lower region. This behaviour is associated with natural convection, where heated air becomes less dense and rises, leading to stratification within the enclosure. This effect is intrinsic to enclosed thermal systems and directly influences the stability of the internal environment.

In addition to vertical stratification, lateral temperature non-uniformities were identified. In Section 1 and Section 2, sensors located on one side of the chamber (positions 3, 6 and 9) exhibited higher temperatures than their counterparts on the opposite side (positions 1, 4 and 7). This asymmetry suggests that airflow distribution is not uniform and is strongly influenced by the positioning and operation of the fans responsible for air recirculation.

An inverse temperature distribution was observed in Section 5, located near the air injection outlets. In this region, sensors positioned at the opposite side (positions 1, 4 and 7) recorded higher temperatures. This behaviour indicates that local airflow patterns and heat input are not symmetrically distributed, potentially due to variations in fan performance or heating element efficiency.

One of the most relevant findings is the identification of regions with comparatively higher thermal stability within the chamber. In this study, thermal stability was defined in relative terms using two quantitative indicators calculated for each section: the spatial temperature range and the spatial standard deviation. Under this criterion, Section 3 was identified as the most stable region because it showed the lowest temperature range, 1.0 °C, and one of the lowest spatial standard deviations, 0.38 °C. Section 4 also remained within a moderate temperature range, although a clear vertical gradient was still observed. Since these sections are located within the main printing region, their comparatively lower thermal variability is relevant for the deposition process. The admissible temperature variation is bioink-specific and depends on the formulation, gelation mechanism and thermal transition range of the material.

The central printing region showed lower thermal variation than the air-recirculation and boundary regions. This indicates that the area where material deposition occurs is exposed to relatively more stable thermal conditions, which may help maintain more consistent bioink properties and support process reproducibility during biofabrication.

**Figure 7:**
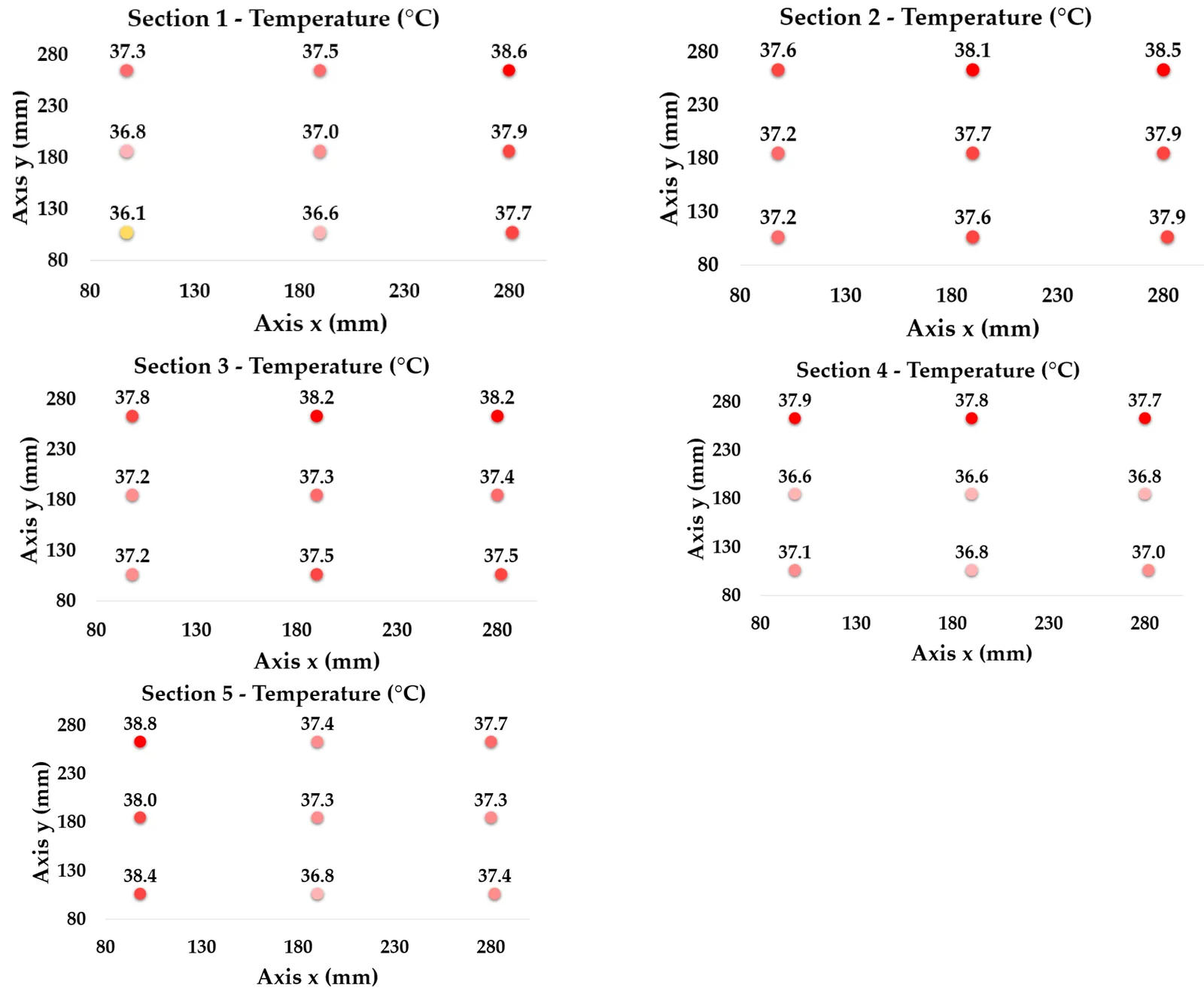
Spatial distribution of the mean temperature values recorded by the sensor array in the five measurement sections of the bioprinting chamber. Each map represents the average temperature obtained at the nine sensor positions after thermal stabilization.

To provide a quantitative comparison of the temperature distribution, the mean temperature values for each sensor position were calculated as described in Section 2.4. The results are shown in Figure 7 and Table 1, where spatial differences between sections can be clearly observed. These data confirm the presence of vertical gradients and localized non-uniformities, reinforcing the combined influence of natural convection and forced airflow on the thermal behaviour of the system.

The reported values correspond to the stabilized thermal regime reached after approximately 45 min from ambient conditions. Therefore, the spatial maps describe the steady operating state of the chamber and not the complete start-up transient.

When interpreting these values, the nominal accuracy of the sensors must be considered (Table 2). Temperature ranges clearly exceeding ±0.5 °C, such as those observed in Section 1 and Section 5, support the presence of relevant spatial non-uniformities within the chamber. In contrast, smaller point-to-point differences should be interpreted cautiously and mainly as relative trends within the limits of the measurement system.

**Table 2:** Statistical analysis of spatial temperature differences among sensor positions in each chamber section.

Section	Friedman χ^2^	df	*p*-Value	Kendall’s W
Section 1	22.81	8	0.0036	0.95
Section 2	23.59	8	0.0027	0.98
Section 3	23.54	8	0.0027	0.98
Section 4	23.57	8	0.0027	0.98
Section 5	23.87	8	0.0024	0.99

The Friedman test showed statistically significant spatial differences among sensor positions in all chamber sections (*p* < 0.01). Kendall’s W ranged from 0.95 to 0.99, indicating a consistent spatial temperature pattern. These results support that the observed thermal differences were not only associated with measurement variability, but reflected reproducible spatial distributions within the chamber.

The observed thermal variations define the environmental conditions to which temperature-sensitive bioinks would be exposed during deposition and initial stabilization. In thermoresponsive formulations, such as gelatin-alginate blends, the maximum temperature range observed in Section 1, 2.5 °C, may be relevant because gelatin-containing systems exhibit temperature-dependent changes in viscosity and physical network formation. Warmer regions within the chamber may reduce viscosity and delay physical stabilization, whereas cooler regions may promote faster gelation or increased resistance to flow. These local differences could affect filament spreading, shape retention and early construct stability, particularly when the bioink formulation operates close to its thermal transition range. For cell-laden formulations, these local temperature differences may modify the matrix microenvironment through changes in gelation kinetics, stiffness and construct stability. This interpretation is consistent with previous studies showing that printing temperature, ambient temperature and bioink temperature influence the extrusion behaviour and final printing outcome of alginate-gelatin hydrogels [11], and with reports linking bioink properties to printability and cell viability [9]. Therefore, the thermal ranges observed in the chamber, particularly in Section 1 and Section 5, indicate that the nominal setpoint alone does not fully describe the actual thermal conditions present within the working volume.

This interpretation is consistent with studies relating bioink rheology to printability. Gao et al. [17] showed that parameters such as the storage modulus, loss modulus and loss factor influence filament uniformity and the structural integrity of printed constructs. Complementarily, Ahmadi Soufivand et al. [18] highlighted that the rheological stability of alginate-gelatin hydrogels is essential for maintaining stable extrusion flow and obtaining multilayer structures with good geometrical fidelity. Therefore, the volumetric characterisation performed in this study allows chamber regions with comparatively more stable thermal conditions to be identified under the experimental reference configuration.

Overall, the experimental results demonstrate that the chamber does not exhibit a fully homogeneous temperature field, but instead presents predictable thermal patterns governed by airflow configuration and heat source distribution. Understanding these patterns is essential for optimizing chamber design and ensuring stable environmental conditions in 3D bioprinting processes.

The observed thermal behaviour is consistent with the dominant heat transfer mechanisms in extrusion-based bioprinting systems, where heat transfer is primarily governed by conduction and convection between the bioink, the printing components and the surrounding air, while radiative effects can be considered negligible [13]. The presence of vertical temperature gradients and localized non-uniformities observed in this study reflects the combined influence of natural convection and forced airflow within the enclosure.

Current approaches for analyzing thermal behaviour in bioprinting systems rely predominantly on coupled fluid-heat models, especially in small-scale or simplified geometries, where the influence of chamber design and material properties on temperature uniformity can be evaluated [19]. However, at the scale of full bioprinting chambers, most studies rely on numerical simulations, while experimental validation is typically limited to discrete sensors or surface measurements [13,19,20]. As a result, the spatial distribution of temperature within the full chamber volume remains insufficiently characterised.

In this context, the present results contribute to addressing this limitation by providing an experimentally based volumetric characterisation of temperature distribution under the defined experimental reference conditions. The identification of both thermal gradients and thermally homogeneous regions highlights the importance of airflow design and heat distribution in achieving stable biofabrication environments.

Although advanced experimental techniques such as fluorescence-based temperature mapping or high-resolution infrared thermography have been successfully applied in microfluidic systems and small-scale devices [21,22,23,24], their application to full-scale bioprinting chambers remains limited. Extending these methodologies to bioprinting environments could provide higher spatial resolution and enable a deeper understanding of heat transfer mechanisms, supporting the optimisation of chamber design and thermal control strategies.

### 3.2. Temperature-Dependent Rheological Response of the Gelatin/DMEM Hydrogel

To assess the practical relevance of the spatial temperature differences measured within the chamber, the temperature-dependent rheological behaviour of the gelatin/DMEM hydrogel was investigated by means of an oscillatory temperature sweep and steady-shear flow measurements.

**Figure 8:**
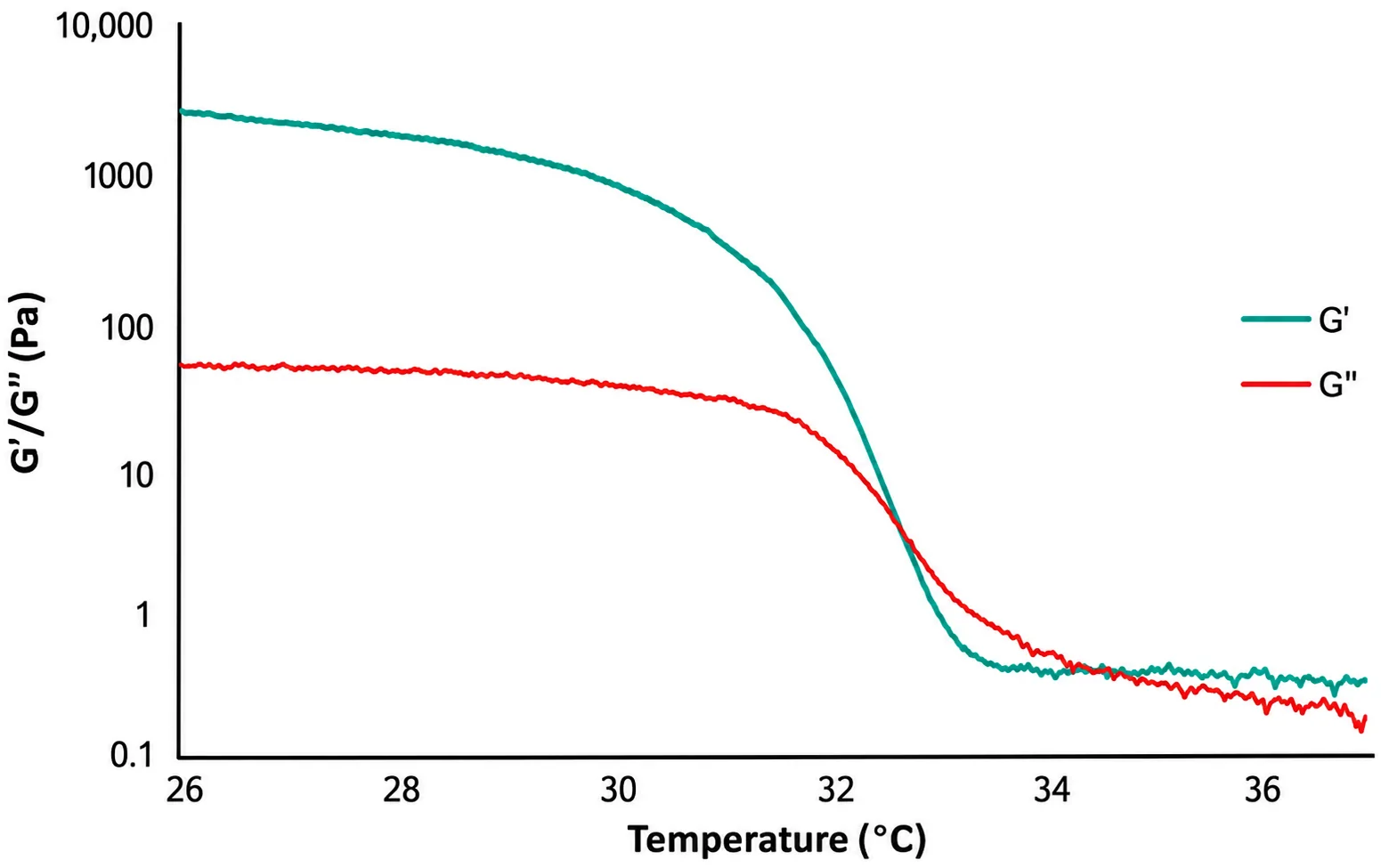
Temperature-dependent viscoelastic response of the gelatin/DMEM hydrogel. Storage modulus (G′) and loss modulus (G″) were recorded during an oscillatory temperature sweep.

The temperature sweep revealed a marked thermal dependence of the viscoelastic response of the formulation (Figure 8). At the lowest temperatures, the storage modulus, G′, remained clearly higher than the loss modulus, G″, indicating a predominantly elastic, gel-like response. Both moduli progressively decreased as the temperature increased, reflecting the gradual weakening of the physical gelatin network. The most pronounced change occurred between approximately 31 and 33 °C, where G′ decreased sharply and approached G″. Above approximately 34 °C, both moduli remained below approximately 1 Pa, indicating that the hydrogel had reached a weakly structured, predominantly fluid-like state.

**Figure 9:**
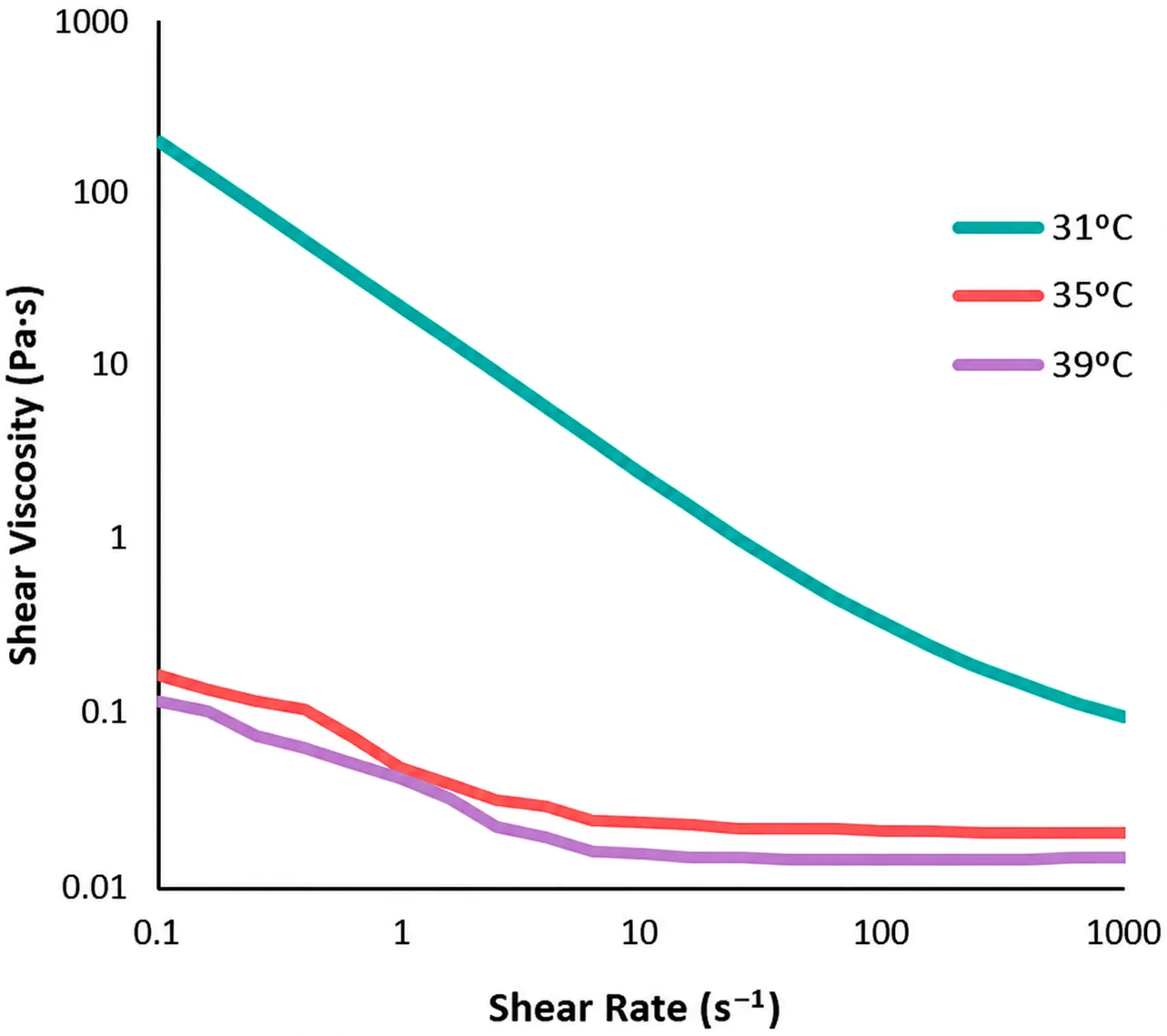
Apparent viscosity measurements of the gelatin/DMEM hydrogel as a function of shear rate at 31, 35, and 39 °C.

The steady-shear measurements further confirmed the temperature sensitivity of the formulation (Figure 9). The apparent viscosity decreased with increasing shear rate at all the temperatures evaluated, demonstrating shear-thinning behaviour. The hydrogel measured at 31 °C exhibited a substantially higher apparent viscosity than that recorded at 35 and 39 °C throughout the analysed shear-rate range. In contrast, the flow curves obtained at 35 and 39 °C were closer to each other, suggesting that the most pronounced rheological change occurred as the material passed through its thermal transition region, rather than occurring uniformly across the entire temperature range [17,25,26].

These results provide a rheological basis for interpreting the relevance of thermal control during the fabrication of multilayer hydrogel constructs. Following extrusion, the deposited filaments must resist both their own spreading and the progressive mechanical load imposed by subsequently deposited layers. A reduction in viscosity and elastic contribution may increase filament relaxation and deformation, thereby decreasing shape retention and the ability of the construct to support its own weight. Previous studies have similarly related the rheological properties of gelatin-containing bioinks, including G′, G″ and apparent viscosity, to filament formation, geometrical fidelity and the stability of multilayer structures [17,26].

In the chamber analysed, the measured operating temperatures were predominantly above the main transition region identified for the gelatin/DMEM formulation. Consequently, the hydrogel would be expected to remain in a mechanically weak state throughout most of the chamber volume. Nevertheless, spatial temperature differences may still contribute to local variations in flow resistance and post-deposition relaxation. During layer-by-layer fabrication, material deposited at different positions or heights may therefore experience slightly different thermal histories, potentially leading to non-uniform filament spreading and the accumulation of deformation within the construct.

Overall, these findings support that temperature control is particularly relevant for thermoresponsive hydrogel bioinks because relatively small thermal variations within a narrow transition region can shift the material from a predominantly elastic, gel-like state to a weakly structured, fluid-like state. This transition is directly relevant to extrusion-based bioprinting, where the same formulation must flow through the nozzle but also recover sufficient mechanical stability after deposition to preserve filament geometry and support subsequent layers. Previous studies have shown that printing temperature, ambient temperature and bioink temperature influence the extrusion behaviour and final printing outcome of alginate-gelatin hydrogels [11], and that rheological parameters such as G′, G″ and apparent viscosity are closely related to filament formation, geometrical fidelity and multilayer stability [17,18]. Therefore, the rheological results provide material-level evidence of the influence of thermal uniformity on the flow behaviour, post-deposition stability and self-supporting capacity of temperature-sensitive bioink formulations.

### 3.3. CFD Visualisation of the Temperature Field with Internal Printer Components

The CFD analysis included two numerical configurations. The reference configuration reproduced the chamber arrangement used during the experimental temperature mapping, whereas the operational configuration incorporated simplified stationary adiabatic representations of the printhead and build platform. The operational configuration therefore reproduced the geometric presence of the principal printer components located within the thermally controlled enclosure.

**Figure 10:**
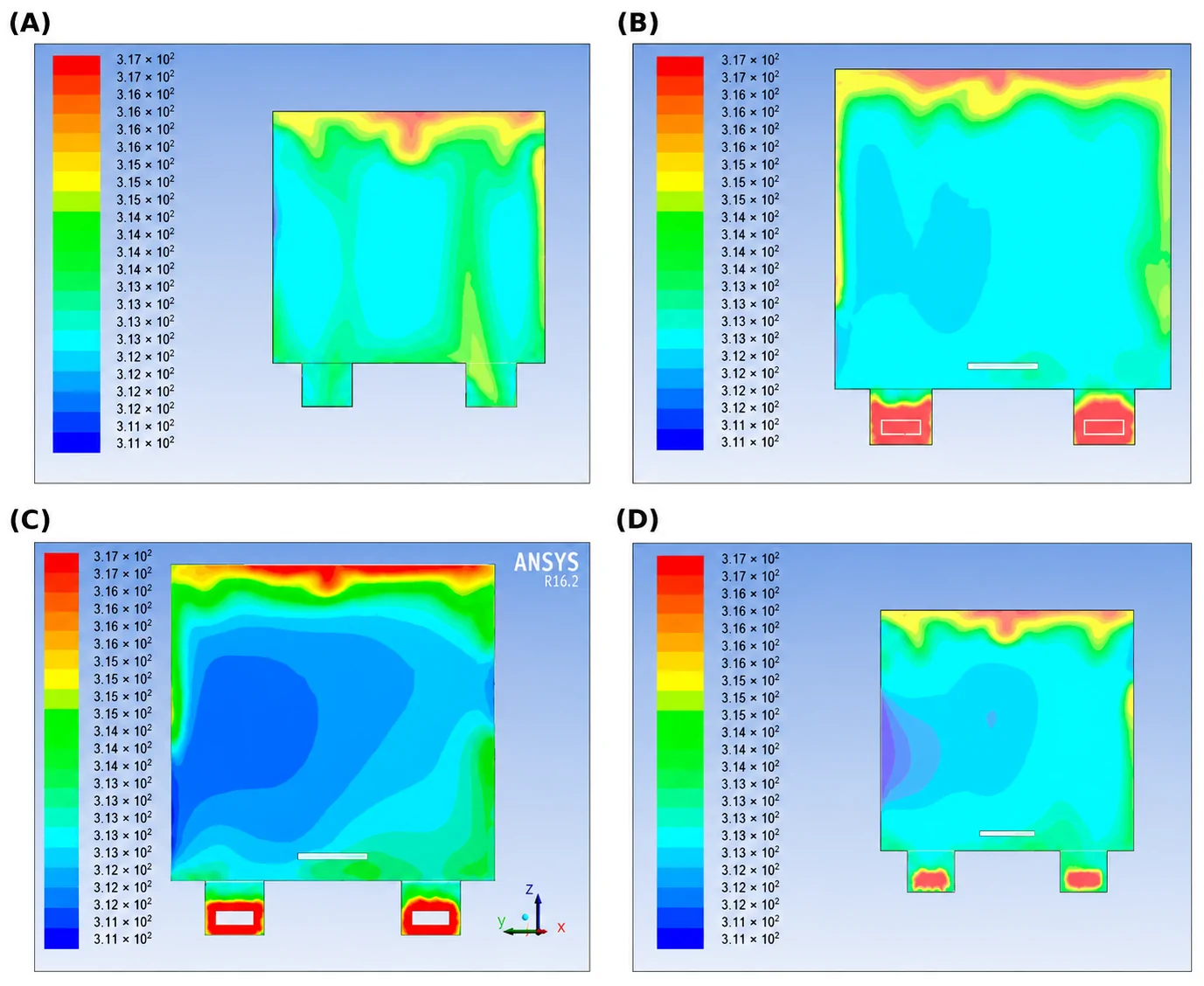
Temperature contours obtained from the reference CFD configuration of the controlled bioprinting chamber. Panels (**A**–**D**) show representative numerical temperature maps used to visualise the principal thermal patterns generated by the chamber geometry, heating system and air-recirculation arrangement. Warmer regions were mainly observed in the upper part of the enclosure and near the thermal-conditioning regions, whereas lateral variations were associated with the internal airflow pathways.

The temperature contours obtained for the reference configuration are shown in Figure 10. Higher-temperature regions were mainly located in the upper part of the chamber and close to the thermal-conditioning and air-recirculation system. The main numerical trend was the formation of a vertical thermal gradient, with higher temperatures in the upper chamber region. This behaviour resulted from the combined action of buoyancy-driven upward air movement and forced recirculation generated by the axial fans.

Lateral variations were also observed in the reference CFD temperature field. The relative positions of the fans, air inlets, outlets and heating elements modified the local circulation pathways, generating heat-accumulation zones and regions with different levels of thermal homogeneity.

**Figure 11:**
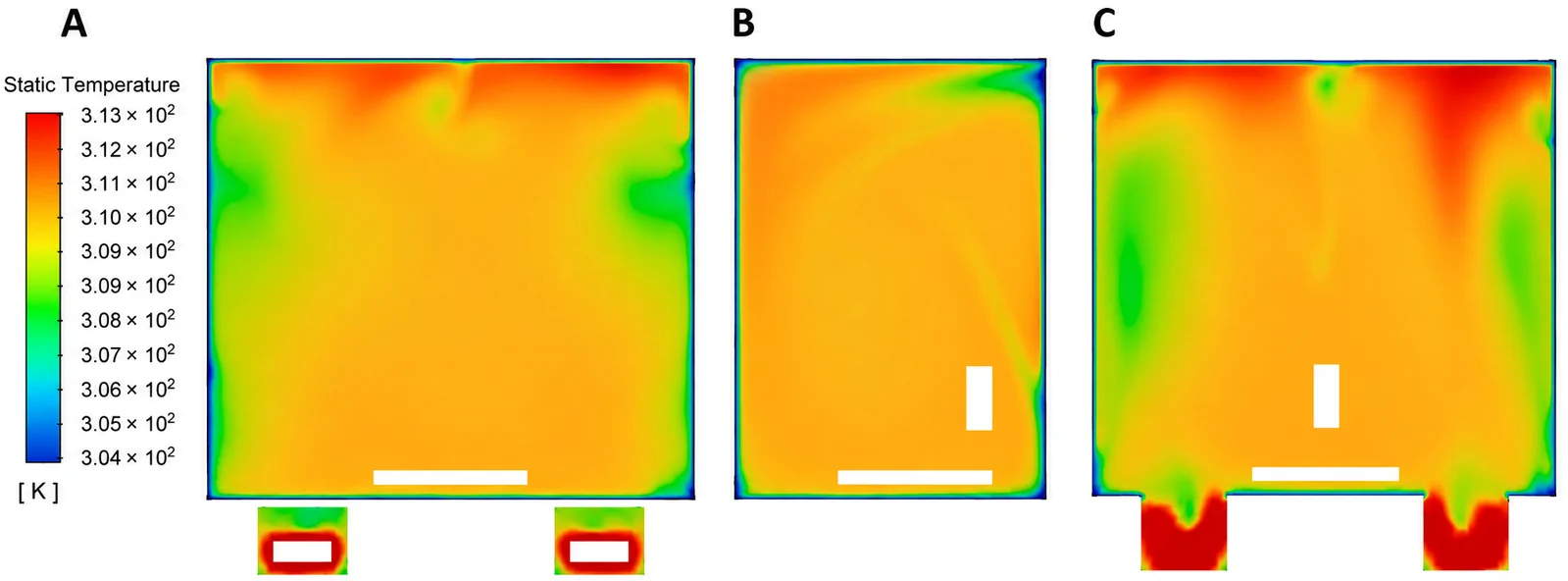
Temperature contours obtained for the operational CFD configuration incorporating the simplified printhead and build platform. Panels (**A**–**C**) show representative sections of the simulated temperature field and illustrate the temperature field in the presence of the internal printer components.

The temperature contours corresponding to the operational configuration, including the printhead and build platform, are shown in Figure 11.

The printhead and build platform acted solely as adiabatic geometric obstacles within the airflow domain. The temperature field around these components therefore reflected the redistribution of the recirculating air caused by their geometric presence, rather than heat storage or thermal conduction through the components.

From a bioprinting perspective, the experimental results identified the central working region as comparatively more stable than the peripheral regions and the areas located close to the recirculation system. The operational CFD configuration provides a visual representation of the temperature field around the printhead and build platform within this working region.

The combined experimental and numerical approach may support future improvements aimed at reducing local heat accumulation and increasing thermal homogeneity within the printing region. Potential modifications include adjusting the position or orientation of air inlets and outlets, improving airflow distribution in the upper part of the chamber, redistributing the heating elements and incorporating additional feedback-control points.

## 4. Conclusions

This study presents a volumetric thermal characterisation of a controlled atmospheric chamber for 3D bioprinting using a multi-point temperature sensing approach. The proposed acquisition system enabled temperature monitoring at 45 spatial locations distributed across five measurement sections, providing a more detailed description of the internal thermal behaviour of the chamber than conventional single-point measurements.

The experimental results showed that the chamber does not present a fully homogeneous temperature field under the analysed operating conditions. Instead, the thermal distribution was characterised by vertical gradients, lateral asymmetries and local non-uniformities associated with natural convection, forced air recirculation and the position of the heating and ventilation elements. These findings highlight the importance of evaluating the full chamber volume rather than relying exclusively on local temperature control points.

From a biofabrication perspective, the central printing region showed relatively more stable thermal conditions than the peripheral and recirculation areas, particularly in Section 3 and, to a lesser extent, Section 4.

The CFD model incorporated the internal air volume, heating and recirculation systems, chamber walls, and stationary adiabatic representations of the printhead and build platform. These representations reproduced the geometric presence of the internal printer components as airflow obstacles and enabled visualisation of the surrounding temperature field.

The combination of experimental volumetric sensing and CFD analysis provides useful information for identifying heat-accumulation zones and guiding future improvements in chamber design. Potential modifications include adjusting the position or orientation of air inlets and outlets, improving airflow distribution in the upper region of the chamber, redistributing heating elements to reduce local hot spots and incorporating additional feedback-control points within the working volume. These strategies could help reduce vertical gradients and lateral asymmetries and improve temperature homogeneity within the printing area.

Overall, this work contributes to the development of more controlled and reproducible biofabrication environments. By improving the understanding of the thermal conditions inside bioprinting chambers, the proposed approach may support the optimisation of next-generation 3D bioprinting platforms intended for the fabrication of functional biomimetic systems.

## Figures and Tables

**Table 1:** Quantitative comparison of the temperature distribution in the five measurement sections of the bioprinting chamber.

Section	Mean Temperature (°C)	Minimum Temperature (°C)	Maximum Temperature (°C)	Temperature Range (°C)	Spatial Standard Deviation (°C)	Main Observation
Section 1	37.3	36.1	38.6	2.5	0.75	Marked lateral asymmetry and vertical gradient
Section 2	37.7	37.2	38.5	1.3	0.40	Moderate thermal variation, with warmer upper/right region
Section 3	37.6	37.2	38.2	1.0	0.38	Lowest overall temperature range among the analysed sections
Section 4	37.2	36.6	37.9	1.3	0.51	Moderate range, but with a clear vertical gradient
Section 5	37.7	36.8	38.8	2.0	0.63	Higher variability near the air injection/recirculation region

## Data Availability

The data generated and analyzed during the current study are available from the corresponding author upon request.
